# QTLs associated with dry matter intake, metabolic mid-test weight, growth and feed efficiency have little overlap across 4 beef cattle studies

**DOI:** 10.1186/1471-2164-15-1004

**Published:** 2014-11-20

**Authors:** Mahdi Saatchi, Jonathan E Beever, Jared E Decker, Dan B Faulkner, Harvey C Freetly, Stephanie L Hansen, Helen Yampara-Iquise, Kristen A Johnson, Stephen D Kachman, Monty S Kerley, JaeWoo Kim, Daniel D Loy, Elisa Marques, Holly L Neibergs, E John Pollak, Robert D Schnabel, Christopher M Seabury, Daniel W Shike, Warren M Snelling, Matthew L Spangler, Robert L Weaber, Dorian J Garrick, Jeremy F Taylor

**Affiliations:** Department of Animal Science, Iowa State University, Ames, 50011 USA; Department of Animal Sciences, University of Illinois, Urbana, 61801 USA; Division of Animal Sciences, University of Missouri, Columbia, 65211 USA; Department of Animal Sciences, The University of Arizona, Tucson, 85719 USA; USDA, ARS, US Meat Animal Research Center, Clay Center, Kragujevac, 68933 USA; Department of Animal Sciences, Washington State University, Pullman, 99164 USA; Department of Statistics, University of Nebraska, Lincoln, 68583 USA; GeneSeek a Neogen Company, Lincoln, 68521 USA; Department of Veterinary Pathobiology, Texas A&M University, College Station, 77843 USA; Department of Animal Science, University of Nebraska, Lincoln, 68583 USA; Department of Animal Sciences and Industry, Kansas State University, Manhattan, 66506 USA; Institute of Veterinary, Animal and Biomedical Sciences, Massey University, Palmerston North, New Zealand

**Keywords:** GWAS, QTL, Genomic selection, Feed efficiency, Beef cattle

## Abstract

**Background:**

The identification of genetic markers associated with complex traits that are expensive to record such as feed intake or feed efficiency would allow these traits to be included in selection programs. To identify large-effect QTL, we performed a series of genome-wide association studies and functional analyses using 50 K and 770 K SNP genotypes scored in 5,133 animals from 4 independent beef cattle populations (Cycle VII, Angus, Hereford and Simmental × Angus) with phenotypes for average daily gain, dry matter intake, metabolic mid-test body weight and residual feed intake.

**Results:**

A total of 5, 6, 11 and 10 significant QTL (defined as 1-Mb genome windows with Bonferroni-corrected P-value <0.05) were identified for average daily gain, dry matter intake, metabolic mid-test body weight and residual feed intake, respectively. The identified QTL were population-specific and had little overlap across the 4 populations. The pleiotropic or closely linked QTL on BTA 7 at 23 Mb identified in the Angus population harbours a promising candidate gene *ACSL6* (acyl-CoA synthetase long-chain family member 6), and was the largest effect QTL associated with dry matter intake and mid-test body weight explaining 10.39% and 14.25% of the additive genetic variance, respectively. Pleiotropic or closely linked QTL associated with average daily gain and mid-test body weight were detected on BTA 6 at 38 Mb and BTA 7 at 93 Mb confirming previous reports. No QTL for residual feed intake explained more than 2.5% of the additive genetic variance in any population. Marker-based estimates of heritability ranged from 0.21 to 0.49 for residual feed intake across the 4 populations.

**Conclusions:**

This GWAS study, which is the largest performed for feed efficiency and its component traits in beef cattle to date, identified several large-effect QTL that cumulatively explained a significant percentage of additive genetic variance within each population. Differences in the QTL identified among the different populations may be due to differences in power to detect QTL, environmental variation, or differences in the genetic architecture of trait variation among breeds. These results enhance our understanding of the biology of growth, feed intake and utilisation in beef cattle.

## Background

Feed costs comprise the majority of beef production costs and the efficiency of feed utilisation has long been recognised for its economic importance in beef cattle [1, 2]. Improvements in the efficiency of feed utilisation would lead to increased economic returns that would influence the entire beef cattle production system [3]. Feed efficiency is also important for social reasons due to environmental concerns about the methane emissions of cattle and because of the perceived competition in land use for producing crops for direct human consumption or for corn- and soybean-based biofuels. It has been reported that more efficient cattle emit lower amounts of methane [4].

The most commonly used measure of feed efficiency has traditionally been feed conversion ratio, which is the ratio of feed consumed to body weight gain. Selection to improve feed conversion ratio has the potential to increase growth rate in young animals because the two traits are genetically correlated [3]. This could, as a correlated response, produce larger females which are more expensive to maintain in the breeding herd [3]. Residual feed intake (RFI) is an alternate measure of feed efficiency, defined as the difference between an animal’s actual and expected feed intake based on its body weight and growth rate during the feeding period [1, 3]. RFI is considered by some to be a preferred method of measuring feed efficiency because of its phenotypic independence from the traits used to calculate RFI [5].

The estimated heritability of RFI in cattle populations is moderate to high, ranging from 0.08 to 0.46 [6–8]. Based on these heritability estimates and substantial phenotypic variation, RFI has the potential for inclusion in selection criteria to improve feed efficiency and the profitability of beef production [3, 9, 10]. It has also been experimentally demonstrated that direct selection on RFI can improve the feed efficiency of cattle [11]. However, individual feed intake measurements are needed for direct selection and these are expensive to obtain. The cost and logistics associated with recording feed intake have historically been the primary limitations to population-wide selection to improve feed efficiency in livestock. This problem could be ameliorated if genetic markers predictive of feed intake or RFI were available. Consequently, there has been considerable recent research to develop genetic markers that can be used to select animals for improved feed efficiency.

Opportunities to identify trait-associated genetic markers have been advanced by the availability of genome-wide high-density panels of single nucleotide polymorphism (SNP) markers including the Illumina BovineSNP50 BeadChip (50 K) [12] and BovineHD BeadChip (770 K) (Illumina Inc., San Diego, CA; [13]). Genome-wide association studies (GWAS) have now identified SNPs associated with economically important traits in both beef and dairy cattle [14–17]. Marker associations with RFI have previously been reported in beef cattle [18–20] and putative quantitative trait loci (QTL) have been mapped to BTA 1, 2, 5, 7, 8, 12, 14, 16, 17, 18, 19, 20, 21, 24, 26, 28 and 29 [19]. Of 8,786 polymorphic SNPs genotyped in 189 Australian beef cattle sampled for either high or low RFI, 161 were trait-associated (P <0.01) [18]. However, only two of these SNPs remained significant when evaluated in a larger multi-breed sample of animals [18].

The simplest model for performing GWAS is linear regression, where the association between markers and a trait of interest is tested one marker at a time. This type of analysis has been used for GWAS in humans [21] and in animal populations [22] where the extent of linkage disequilibrium among markers is considerably greater [23, 24] due to the small effective population sizes of most livestock breeds. On the other hand, Bayesian variable selection models facilitate the simultaneous fitting of all markers in the model and have been used for GWAS in livestock [25–31] to improve the precision of QTL mapping [32]. Among several Bayesian variable selection models, BayesB [33] has been shown by simulation to map QTL more precisely than other methods [34]. BayesB has also been shown to implicitly account for the population stratification resulting from pedigree relationships [35].

Although several QTL associated with feed efficiency traits in beef cattle have been reported, not all of the genetic variation in these traits has been captured because of inadequate sample size or studies limited to a single population. The extent of genetic variation for feed efficiency traits among different beef cattle populations remains unexplored. The objectives of this study were to map QTL associated with feedlot RFI and its growth and feed intake components; specifically, average daily gain on feed (ADG; kg/d), average daily dry matter intake (DMI; kg/d) and mid-test metabolic body weight (MBW; kg^0.75^) in a relatively large sample of animals (N =5,133) from 4 different beef cattle populations (Cycle VII, Angus, Hereford and Simmental × Angus, see Methods for more details). A BayesB model was used to simultaneously analyse SNP markers and identify QTL by characterising the proportion of additive genetic variation explained by every non-overlapping 1-Mb region within the genome.

## Results and Discussion

### Posterior means of additive genetic and residual variances and heritability

For each trait, the analysis generates an estimate of the proportion of phenotypic variation that can be explained by the use of SNP markers to represent identity by state among individuals, which is similar to the heritability estimated when pedigree information is used to represent identity by descent among individuals. The posterior means of heritability, additive genetic and residual variances for ADG, DMI, MBW and RFI in each of the 4 populations (Cycle VII, Angus, Hereford and Simmental × Angus) are in Table 1. Estimates of heritability ranged from 0.19 to 0.30 for ADG, from 0.27 to 0.41 for DMI, from 0.38 to 0.50 for MBW, and from 0.21 to 0.49 for RFI and are similar to those reported in the literature [16, 36, 37]. Heritability estimates in the Cycle VII population reported by [37] using a BayesC model were 0.24, 0.41, 0.58 and 0.57 for ADG, DMI, MBW and RFI, respectively which are similar to those produced in this study by applying a BayesB model to the same population (Table 1). The highest estimates of heritability were obtained for the Cycle VII and Hereford populations. The lower heritability estimates obtained for the other populations is likely due to the nutritional trials that were superimposed on animals during the feeding period that resulted in much larger numbers of contemporary groups (see Methods). The moderate to high heritabilities estimated for RFI indicates a significant potential for the identification of QTL considering the available sample sizes in this study.

**Table 1:** **Marker-based estimates of heritability (h** ^**2**^ **), additive genetic variance (V**_**A**_ **) and residual variance (V**_**E**_ **) for ADG, DMI, MBW and RFI in the Cycle VII, Angus, Hereford and Simmental × Angus populations** ^**1**^

Trait	Cycle VII			Angus			Hereford			Simmental × Angus		
	h ^2^	V _A_	V _E_	h ^2^	V _A_	V _E_	h ^2^	V _A_	V _E_	h ^2^	V _A_	V _E_
ADG (kg/d)	0.30	0.01	0.03	0.19	0.01	0.05	0.27	0.02	0.05	0.23	0.01	0.03
DMI (kg/d)	0.35	0.39	0.71	0.35	0.85	1.55	0.41	0.66	0.94	0.27	0.28	0.75
MBW (kg^0.75^)	0.47	25.73	29.49	0.49	38.08	39.78	0.50	24.02	23.79	0.38	8.58	14.17
RFI (kg/d)	0.49	0.19	0.19	0.21	0.27	0.98	0.45	0.32	0.40	0.32	0.20	0.42

^1^ADG: average daily gain, DMI: dry matter intake, MBW: mid-test metabolic body weight, and RFI: residual feed intake.

### Genome wide association – general results

**Figure 1:**
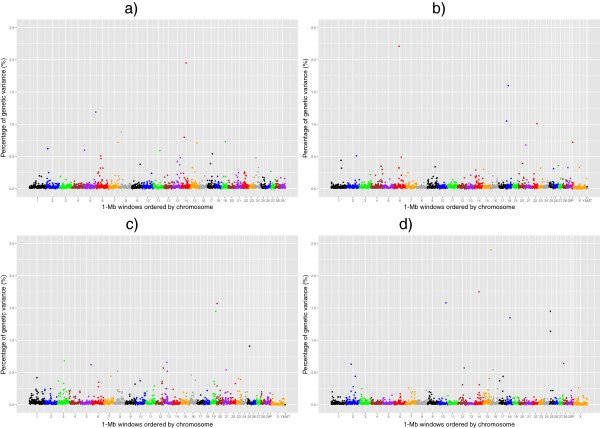
**The residual feed intake QTL.** Proportion of additive genetic variance explained by SNPs within each 1-Mb window across the genome for residual feed intake in 4 different beef populations: **a)** Cycle VII, **b)** Angus, **c)** Hereford and **d)** Simmental × Angus. P: Pseudo autosomal region on BTAX, MT: Mitochondrial DNA. Based on UMD3.1 and Y chromosome assembly from Btau4.6.1.

**Figure 2:**
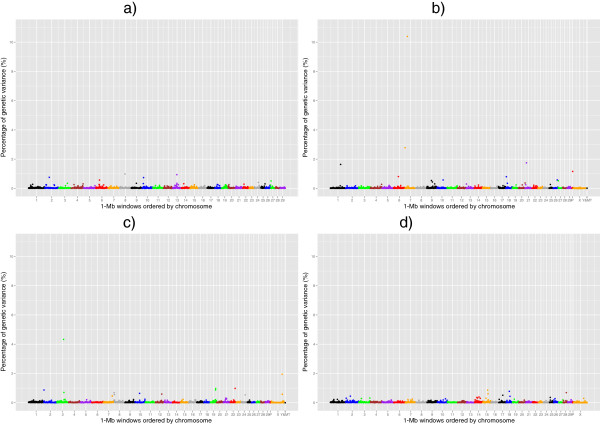
**The feedlot dry matter intake QTL.** Proportion of additive genetic variance explained by SNPs within each 1-Mb window across the genome for feedlot dry matter intake in 4 different beef populations: **a)** Cycle VII, **b)** Angus, **c)** Hereford and **d)** Simmental × Angus. P: Pseudo autosomal region on BTAX, MT: Mitochondrial DNA. Based on UMD3.1 and Y chromosome assembly from Btau4.6.1.

**Figure 3:**
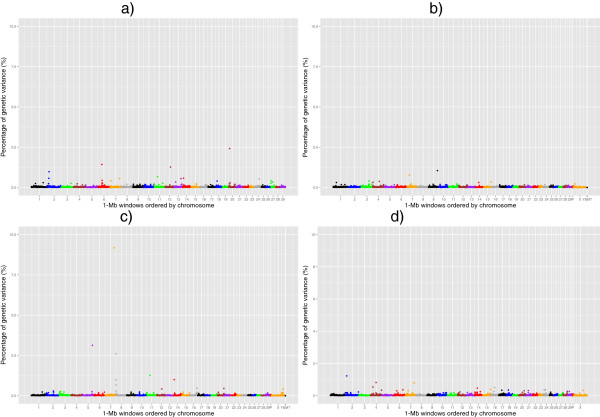
**The feedlot average daily gain QTL.** Proportion of additive genetic variance explained by SNPs within each 1-Mb window across the genome for feedlot average daily gain in 4 different beef populations: **a)** Cycle VII, **b)** Angus, **c)** Hereford and **d)** Simmental × Angus. P: Pseudo autosomal region on BTAX, MT: Mitochondrial DNA. Based on UMD3.1 and Y chromosome assembly from Btau4.6.1.

**Figure 4:**
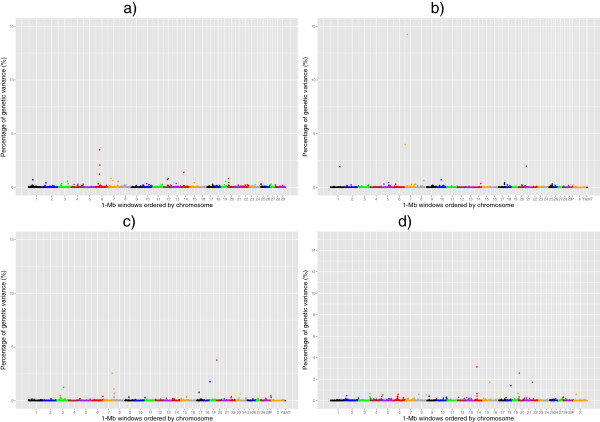
**The mid-test metabolic body weight QTL.** Proportion of additive genetic variance explained by SNPs within each 1-Mb window across the genome for mid-test metabolic body weight in 4 different beef populations: **a)** Cycle VII, **b)** Angus, **c)** Hereford and **d)** Simmental × Angus. P: Pseudo autosomal region on BTAX, MT: Mitochondrial DNA. Based on UMD3.1 and Y chromosome assembly from Btau4.6.1.

Manhattan plots of the posterior means of the additive genetic variance explained by each 1-Mb window across the genome for RFI, DMI, ADG and MBW are in Figures 1, 2, 3 and 4, respectively. The numbers of chromosome segments shown in Figures 1, 2, 3 and 4 (the X-axis) are not the same across the different populations as different SNP genotyping platforms were used and slightly different SNP filtering criteria were utilised in the different populations (See Methods). Some 1-Mb windows with a Bonferroni-corrected P-value less than 0.05 were identified as significant QTL and are summarised in Tables 2, 3, 4 and 5 for RFI, DMI, ADG, and MBW, respectively. The identity of the most strongly associated SNP (denoted throughout as ‘lead-SNP’ and defined as the SNP with the highest posterior probability of inclusion (sPPI) within the significant 1-Mb window) is also reported for each QTL.

**Table 2:** **Large-effect QTL associated with residual feed intake in 4 different beef populations**

BTA_Mb ^1^	Population ^2^	Start SNP	End SNP	Number of SNPs	Genetic variance (%)	Nominal P-value	Bonferroni corrected P-value	Lead-SNP	sPPI ^3^
6_50	Angus	*rs133728493*	*rs136948693*	304	2.21	1.10E^−6^	2.95E^−3^	*rs137524648*	0.04
10_85	Sim × Ang	*rs110164488*	*rs43652141*	230	1.58	7.37E^−6^	1.97E^−2^	*rs136969055*	0.06
14_41	Sim × Ang	*rs42509065*	*rs133984439*	201	1.75	4.17E^−6^	1.12E^−2^	*rs136041102*	0.31
14_43	Cycle VII	*rs109845775*	*rs110706635*	12	1.95	2.26E^−6^	5.70E^−3^	*rs41617069*	0.70
15_82	Sim × Ang	*rs110524424*	*rs42781637*	380	2.40	6.78E^−7^	1.81E^−3^	*rs41785720*	0.05
18_22	Angus	*rs41579995*	*rs132921208*	292	1.60	6.88E^−6^	1.85E^−2^	*rs109634056*	0.07
18_37	Sim × Ang	*rs110857287*	*rs43211307*	241	1.35	1.74E^−5^	4.65E^−2^	*rs137177006*	0.03
19_54	Hereford	*rs134654442*	*rs110630064*	353	1.45	1.18E^−5^	3.17E^−2^	*rs109988749*	0.21
20_4	Hereford	*rs134565601*	*rs43094976*	299	1.57	7.64E^−6^	2.05E^−2^	*rs133032375*	0.05
25_7	Sim × Ang	*rs110477162*	*rs110037478*	289	1.45	1.18E^−5^	3.16E^−2^	*rs137078861*	0.04

^1^Bovine chromosome and n^th^ 1-Mb window within the same chromosome starting at 0 Mb and based on the UMD3.1 assembly.

^2^Sim × Ang stands for Simmental × Angus. The Cycle VII population was genotyped with the BovineSNP50 assay while the other populations were genotyped with the BovineSNP50 and BovineHD assays and imputed to the BovineHD content using Beagle 4.0.

^3^sPPI: posterior probability of inclusion for the given lead-SNP.

**Table 3:** **Large-effect QTL associated with feedlot dry matter intake in 4 different beef populations**

BTA_Mb ^1^	Population	Start SNP	End SNP	Number of SNPs	Genetic variance (%)	Nominal P-value	Bonferroni corrected P-value	Lead-SNP	sPPI ^2^
1_107	Angus	*rs137640861*	*rs133218870*	210	1.64	1.52E^−5^	4.08E^−2^	*rs136742116*	*0.*10
3_70	Hereford	*rs134410518*	*rs136347800*	195	4.33	7.23E^−8^	1.94E^−4^	*rs109239108*	0.14
7_0	Angus	*rs134214229*	*rs133987755*	219	2.78	9.14E^−7^	2.45E^−3^	*rs134458731*	0.49
7_23	Angus	*rs133100477*	*rs42926834*	261	10.39	2.97E^−10^	7.97E^−7^	*rs133232710*	0.44
21_13	Angus	*rs109890770*	*rs137407067*	277	1.75	1.09E^−5^	2.92E^−2^	*rs134953219*	*0.31*
X_115	Hereford	*rs109289869*	*rs133784615*	214	1.95	6.20E^−6^	1.66E^−2^	*rs134244037*	0.16

^1^Bovine chromosome and n^th^ 1-Mb window within the same chromosome starting at 0 Mb and based on the UMD3.1 assembly.

^2^sPPI: posterior probability of inclusion for the given lead-SNP.

**Table 4:** **Large-effect QTL associated with feedlot average daily gain in 4 different beef populations** ^**1**^

BTA_Mb ^1^	Population ^2^	Start SNP	End SNP	Number of SNPs	Genetic variance (%)	Nominal P-value	Bonferroni corrected P-value	Lead-SNP	sPPI ^3^
5_106	Hereford	*rs135296291*	*rs137324049*	312	3.13	1.37E^−7^	3.67E^−4^	*rs132862617*	0.09
6_38	Cycle VII	*rs29010895*	*rs81131471*	21	1.43	1.27E^−5^	3.21E^−2^	*rs109294917*	0.36
7_93	Hereford	*rs134145330*	*rs109802727*	183	9.18	1.07E^−10^	2.87E^−7^	*rs109618368*	0.11
8_0	Hereford	*rs133933459*	*rs134191169*	287	2.60	4.21E^−7^	1.13E^−3^	*rs136695610*	0.07
20_8	Cycle VII	*rs110676036*	*rs41638185*	25	2.42	6.45E^−7^	1.63E^−3^	*rs42602138*	0.6

^1^Bovine chromosome and n^th^ 1-Mb window within the same chromosome starting at 0 Mb and based on the UMD3.1 assembly.

^2^The Cycle VII population was genotyped with the BovineSNP50 assay while the other populations were genotyped with the BovineSNP50 and BovineHD assays and imputed to the BovineHD content using Beagle 4.0.

^3^sPPI: posterior probability of inclusion for the given lead-SNP.

**Table 5:** **Large-effect QTL associated with mid-test body weight in 4 different beef populations**

BTA_Mb ^1^	Population ^2^	Start SNP	End SNP	Number of SNPs	Genetic variance (%)	Nominal P-value	Bonferroni corrected P-value	Lead-SNP	sPPI ^3^
1_98	Angus	*rs41638981*	*rs110173036*	265	1.94	1.07E^−5^	2.88E^−2^	*rs135605472*	0.55
6_38	Cycle VII	*rs29010895*	*rs81131471*	21	3.49	4.78E^−7^	1.21E^−3^	*rs109294917*	0.55
6_39	Cycle VII	*rs81139192*	*rs81129153*	25	2.08	7.52E^−6^	1.89E^−2^	*rs110012183*	0.54
7_0	Angus	*rs134214229*	*rs133987755*	219	3.99	2.26E^−7^	6.08E^−4^	*rs134458731*	0.89
7_23	Angus	*rs133100477*	*rs42926834*	261	14.24	8.99E^−11^	2.41E^−7^	*rs136491020*	0.57
7_93	Hereford	*rs134145330*	*rs109802727*	183	2.55	2.61E^−6^	7.00E^−3^	*rs110680622*	0.05
14_24	Sim × Ang	*rs109637592*	*rs109636480*	192	3.14	8.54E^−7^	2.29E^−3^	*rs134751608*	0.08
18_63	Hereford	*rs110348373*	*rs42522614*	225	1.76	1.75E^−5^	4.69E^−2^	*rs41897307*	0.39
20_4	Hereford	*rs134565601*	*rs43094976*	299	3.78	3.07E^−7^	8.23E^−4^	*rs133032375*	0.08
20_6	Sim × Ang	*rs42517095*	*rs42352270*	288	2.58	2.45E^−6^	6.56E^−3^	*rs133488748*	0.07
21_13	Angus	*rs109890770*	*rs137407067*	277	1.97	9.92E^−6^	2.66E^−2^	*rs41592029*	0.54

^1^Bovine chromosome and n^th^ 1-Mb window within the same chromosome starting at 0 Mb and based on the UMD3.1 assembly.

^2^Sim × Ang stands for Simmental × Angus. The Cycle VII population was genotyped with the BovineSNP50 assay while the other populations were genotyped with the BovineSNP50 and BovineHD assays and imputed to the BovineHD content using Beagle 4.0.

^3^sPPI: posterior probability of inclusion for the given lead-SNP.

The significant QTL were generally population-specific and had little overlap in genomic location across the 4 beef cattle populations. Although this result could be due to different genetic architectures underlying trait variation among these populations, it may also be due to differences in the power to detect QTL due to the larger numbers of contemporary groups in some populations (202 in Simmental × Angus and 102 in Angus versus 10 and 15 in Hereford and Cycle VII, respectively) or due to gene-by-environment or epistatic interactions. The populations exposed to the large number of different nutritional trials (such as forage feeding, concentrate rations or amino acid and mineral supplementation) were bred in a diverse geographical area (throughout the Midwest United States) in several different years. While genotype-by-environment interactions have been extensively detected using classical quantitative genetic approaches, little has been done to study this phenomenon at the level of the genome. We were not able to test for the existence of gene-by-environment interactions because of a lack of suitable connections between individuals in different contemporary groups across the different populations. Further studies employing special experimental designs are needed to investigate the existence of gene-by-environment interactions for feed efficiency traits in beef cattle.

The QTL on BTA 14 at 24 Mb (associated with MBW in Simmental × Angus, Table 5) was the only QTL that was identified as a suggestive QTL in another population (Cycle VII animals with a nominal P-value of 9.91E^−5^). Two different lead-SNPs (*rs42646660* and *rs134751608*) were model-selected to tag this QTL. The *rs42646660* SNP is located within an intron of *XKR4* (XK, Kell blood group complex subunit-related family, member 4) and *rs134751608* is 0.06 Mb centromeric of *XKR4*. Significant associations have previously been reported between *XKR4* variants and subcutaneous rump fat thickness in cattle [16, 38]. The *PLAG1* (pleiomorphic adenoma gene 1) gene which is located near the 24–25 Mb window boundary on BTA 14 has been shown to have large effects on stature in a Holstein × Jersey F_2_ cross [39] and on carcass weight in Japanese Black cattle [40]. Whether mutations in *XKR4* or in nearby *PLAG1* cause variation in MBW in the Cycle VII and Simmental × Angus animals warrants further investigation.

### Genome wide association results for RFI

Ten significant 1-Mb SNP windows located on 8 different autosomes were detected for RFI (Table 2). The significant QTL separated by 2 Mb on BTA 14 could easily represent the same QTL as the effects were detected in two different populations (Simmental × Angus and Cycle VII). The largest effect 1-Mb SNP window for RFI was detected at 82 Mb on BTA 15 in the Simmental × Angus population and explained 2.40% of the total additive genetic variance with a Bonferroni-corrected P-value threshold of 1.81E^−3^ (Table 2). No QTL associated with RFI has previously been reported in this region of the cattle genome but several QTL associated with body size and production traits have been reported [41, 42].

Among the model-selected lead-SNPs tagging RFI QTL, *rs109988749* located on BTA 19 is approximately 100 bp from the *DNAH17* (dynein, axonemal, heavy chain 17) transcription start site and *rs137078861* located on BTA 25 is within an intron of *C25H16orf72* (chromosome 25 open reading frame, human *C16orf72*), which encodes an as yet uncharacterised protein. The remaining lead-SNPs are intergenic variants. *DNAH17* encodes axonemal dynein [43]. Dyneins are motor protein complexes that use ATP to generate force and movement on microtubules in a wealth of biological processes, including ciliary beating, cell division and intracellular transport [44]. Therefore, mutations which reduce the efficiency of ATP conversion into movement are highly likely to reduce the efficiency of conversion of feed energy intake into maintenance and growth. Furthermore, serious human diseases arise from motor protein dysfunction supporting the potential for large phenotypic effects due to mutations in motor protein genes [45].

**Figure 5:**
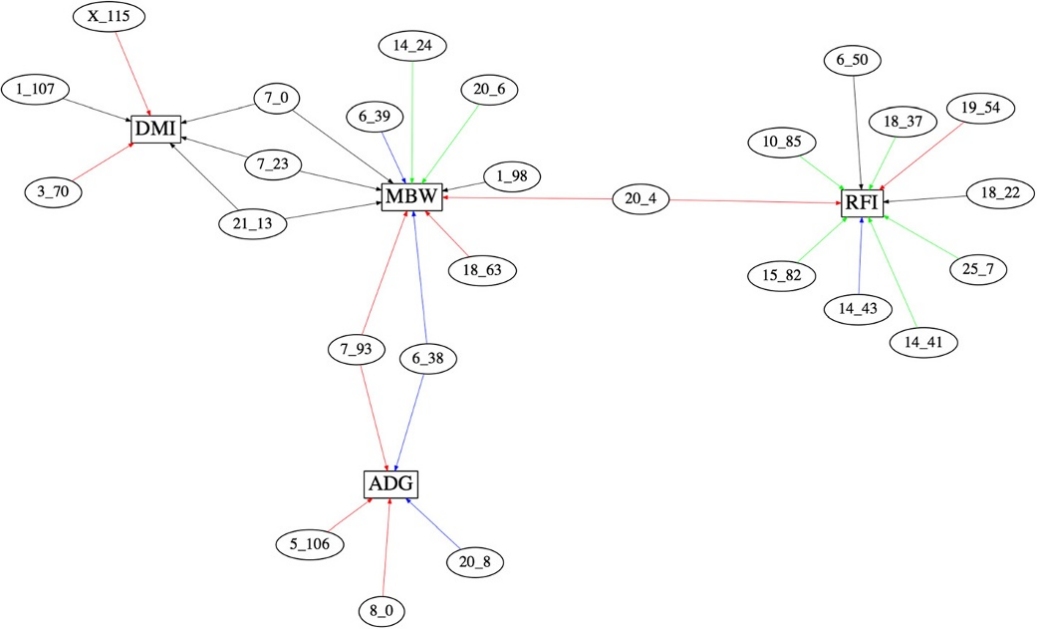
**The QTL network.** The genomic locations (BTA_Mb) and the trait(s) associated with each identified QTL. The traits are average daily gain (ADG), dry matter intake (DMI), metabolic mid-test body weight (MBW) and residual feed intake (RFI). QTL identified in Angus, Cycle VII, Hereford and Simmental × Angus are shown with black, blue, red and green arrows.

Several of the identified QTL possessed pleiotropic effects (Figure 5). In this study, a 1-Mb QTL that was associated with more than one trait was considered to represent a pleiotropic QTL. However, intervals of this size could easily harbour two different closely linked QTL. Further analyses using multivariate models are needed to dissect pleiotropic QTL from closely linked QTL (see [46]). Among these, the QTL on BTA 20 at 4 Mb was the only pleiotropic or closely linked QTL associated with RFI and MBW identified in the Hereford population. While the phenotypic correlations between RFI and its growth and feed intake component traits are expected to be zero, weak genetic correlations exist and pleiotropic loci affecting both traits have previously been reported [17]. We found that the same lead-SNP (*rs133032375*), which is located within an intron of *STC2* (stanniocalcin 2) was selected for both RFI and MBW. The overexpression of human *STC2* in transgenic mice reduces intramembranous and endochondral bone development and skeletal muscle growth and results in a dwarf phenotype [47]. *STC2* has also been shown to be a potent negative regulator of postnatal growth in wild-type mice [48]. While *STC2* is expressed in developing avian striated muscle and joints [49], the physiological roles of *STC2* in cattle are unknown.

### Large-effect pleiotropic or closely linked QTL for DMI and MBW

Three pleiotropic or closely linked QTL on BTA 21 at 13 Mb and BTA 7 at 0 and 23 Mb were associated with DMI and MBW. Lead-SNP *rs134458731* was selected for both DMI and MBW as tagging the QTL on BTA 7 at 0 Mb in Angus (Tables 3 and 5). This SNP lies within an intron of *LOC100125913*, which encodes an uncharacterised protein. The B allele (from the Illumina A/B calling system) at this SNP was at a frequency of 0.09 and was associated with an increase in both DMI and MBW in Angus. The direction of allelic effects at this locus is consistent with the positive genetic correlation between DMI and MBW in cattle.

The pleiotropic or closely linked QTL on BTA 7 at 23 Mb, identified in Angus, was the largest-effect QTL identified for either DMI or MBW and explained 10.39% and 14.25% of the additive genetic variance in each trait, respectively (Tables 3 and 5). Two different lead-SNPs (*rs133232710* and *rs136491020*) were selected for this QTL, with both being within the largest intron of *ACSL6* (acyl-CoA synthetase long-chain family member 6). *ACSL6* is a member of the long-chain acyl-CoA synthetase gene family (ACSL). In mammals, ACSL genes are necessary for fatty acid degradation (β-oxidation), phospholipid remodeling, and the production of long-chain acyl-CoA esters that regulate various physiological, metabolism and cell signaling processes [50, 51]. The ACSL enzymes are membrane-bound, act on non-polar hydrophobic substrates (fatty acids) and their water-soluble and powerful detergent products (acyl-CoAs) are important intermediates for *de novo* lipid synthesis [52]. In the first step of the two-step reaction catalysed by these enzymes, an acyl-AMP intermediate is formed from ATP. AMP is then exchanged with CoA to produce activated acyl-CoA. Once activated, the fatty acid can function as a signalling molecule, be incorporated into phospholipids or triacylglycerides, or undergo β-oxidation in mitochondria for energy generation [52]. While the classical hypothesis in the regulation of feed intake is that a decrease in glucose utilisation registered by specific sensors in the brain leads to hunger, it has also been shown that lipids have an important role through their provision of energy for cell metabolism [53]. Treating mice with fatty acid synthase inhibitors reduces food intake and body weight [54] and increasing neuronal long-chain acyl-CoA in the rat provides a hypothalamic signal of nutrient availability which results in the inhibition of both food intake and glucose production [55]. It has been observed that feeding high fat diets often results in decreased feed and energy intakes in cattle [56], however, the mechanisms that mediate fat-induced depression of feed intake have not been fully investigated in ruminants [57]. Glucose signaling does not effectively regulate feed intake in ruminants [58] because most of the dietary carbohydrates are fermented in the rumen by microorganisms [59]. Consequently, mechanisms other than glucose signaling must control feed intake in cattle and we identify a role for *ACSL6* in this process.

### ADG and MBW QTL on BTA 6 and 7 localise to genomic regions harbouring previously reported pleiotropic QTL

Two pleiotropic or closely linked QTL associated with ADG and MBW were identified on BTA 6 at 38 Mb and BTA 7 at 93 Mb (Figure 5). Many cattle studies have reported QTL on BTA 6 at 38 Mb affecting body weights, growth and carcass traits [60–64], calving ease direct [65], milk traits [66–68], reproductive traits [69–71] and feed efficiency traits [37, 62, 63]. In an analysis of BovineSNP50 genotypes scored in 18,274 animals from 10 US beef cattle breeds with observations for twelve body weights, calving ease and carcass traits, the QTL on BTA 6 at 38 Mb had the largest-effect and was associated with the greatest number of traits in the greatest number of breeds [46]. Three genes have been suggested as positional candidates: *LAP3* (leucine aminopeptidase 3) [68], *NCAPG* (non-SMC condensing I complex, subunit G) [72, 73] and *LCORL* (ligand dependent nuclear receptor corepressor-like) [74]. Lead-SNP *rs109294917*, which lies within an intron of *LCORL*, was selected as tagging the QTL in the analyses of both ADG and MBW.

The pleiotropic QTL on BTA 7 at 93 Mb associated with ADG and MBW was the largest effect QTL identified for ADG and explained 9.18% of the additive genetic variance in Hereford (Table 4). This QTL has previously been shown to be the second largest-effect QTL associated with body weights (birth, weaning, yearling and mature weights) in beef cattle and is segregating in many breeds [46]. Two different intergenic lead-SNPs were selected for this QTL in the analyses of ADG and MBW and both are telomeric of *ARRDC3* (arresting domain containing 3). *ARRDC3* is a member of the arrestin superfamily that regulates obesity in mice and human males [75, 76]. Arrestins are signalling proteins that control metabolism usually through the desensitisation of beta-adrenergic receptors, which are present on the surface of almost every type of mammalian cell. The oral administration of some beta-adrenergic agonists increases muscle and decreases fat accretion in cattle, pigs, poultry, and sheep [77, 78].

The QTL on BTA 5 at 106 Mb explained 3.13% of the additive genetic variance in ADG in Hereford. This QTL appears to be Hereford-specific and pleiotropic, accounting for 2.6, 2.0, 4.9 and 3.9% of the additive genetic variance in birth, weaning, yearling and mature weights, respectively, in an independent population of 2,779 Herefords [46]. The model selected lead-SNP *rs132862617* lies within an intron of *CCND2* (cyclin D2), a member of the family of D-type cyclins which are implicated in cell cycle regulation, differentiation, and oncogenic transformation by governing the activity of cyclin-dependent kinases [79, 80]. Overexpression of *CCND2* in myeloid cells results in a decrease in the duration of G1 (Gap 1 phase in the cell cycle when cell size increases) and an increase in the percentage of cells in S phase (Synthesis phase when DNA replication occurs) in mammalian cells [81, 82]. Since cell proliferation is an essential element of body growth, *CCND2* appears to be a viable candidate gene for this QTL.

## Conclusions

Although many QTL associated with feed efficiency traits of beef cattle have been identified, very little of the genetic variation in these traits has been explained by the detected QTL because of small sample sizes and the fact that the majority of variation appears to be due to small-effect loci. In this study, we took advantage of a relatively large sample size (5,133 animals from 4 independent beef cattle populations) to identify novel QTL and improve the resolution of the location of previously mapped QTL. This study is the largest GWAS ever performed to identify markers associated with feed efficiency and its component traits in beef cattle and led us to discover several large-effect QTL that cumulatively account for a significant percentage of additive genetic variance (the percentages in each of Tables 2 through 5 are additive within a population). Our results also suggest that QTL associated with feed efficiency traits tend to be population-specific with little overlap across populations, which could be due to differences in the power to detect QTL, environmental variation, or differences in the genetic architecture of trait variation among populations. These results also suggest candidate genes for the detected large-effect QTL which will improve our understanding of the biology of growth, feed consumption and feed utilisation in beef cattle.

## Methods

The US Meat Animal Research Center Animal Care and Use Committee approved the procedures used in the experiment applied on Cycle VII animals. For the other 3 experiments data were either collected by commercial producers or were collected under the approval of the University of Missouri (ACUC Protocol 7505) or University of Illinois at Champaign-Urbana (IACUC Protocols 06091 and 09078) Animal Care and Use Committees.

### Animals, phenotypic and genotypic data

Feedlot ADG, daily DMI, and MBW traits were measured in 4 different beef cattle populations. In all cases, average daily gain was estimated as the regression of all available weights on weigh dates and average daily feed intake was estimated for each animal from the daily recorded intake of each animal averaged across the number of days on feed and converted to a dry matter intake based upon the estimated moisture content of the ration. The sampled populations included:

1. Cycle VII: 1,160 F_1_ × F_1_ steers derived from Cycle VII of the US Meat Animal Research Center Germplasm Evaluation Project. A description of the breed composition and mating design for these animals is in [37]. Briefly, in Cycle VII, Angus, Hereford, Simmental, Gelbvieh, Limousin, Red Angus, and Charolais sires were mated to Angus, Hereford and MARC III composite (1/4 Angus, 1/4 Hereford, 1/4 Pinzgauer, 1/4 Red Poll) cows. The resulting F_1_ animals were mated to generate 4-way cross progeny which were individually measured for growth and feed intake. A total of 15 contemporary groups formed using year and season of feeding were represented in these data.
2. Angus: 1,658 Angus steers were produced by breeding registered Angus bulls to commercial cows at the Circle A Ranch in Iberia, MO (N =527), at the MFA Incorporated (N =224), Iowa State University (N =41), or were sourced from producers located throughout Missouri (N =866). Animals were produced over 10 years including in 1999 (N =94), 2000 (N =96), 2001 (N =166), 2003 (N =171), 2004 (N =81), 2005 (N =119), 2008 (N =41), 2010 (N =191), 2011 (N =421) and 2012 (N =278). A total of 173 bulls were identified as having sired 1,057 of the steers with half-sib groups ranging in size from 1 to 81 and averaging 6.1 steers. The remaining 601 steers had unknown sires. Animals were fed commercial concentrate rations at the Circle A Ranch using a Calan Gate feeding system or at the University of Missouri using a GrowSafe system for between 60 and 169 days (60–69 d, N =129; 70–79 d, N =66; 80–89 d, N =695; 108 d, N =89; 112 d, N =94; 120–129 d, N =171; 130–139 d; N =173; 140–149 d, N =191; 169 d, N =50). Weights were taken on a range from 3 to 13 occasions (3, N =445; 4, N =82; 6, N =145; 7, N =102; 8, N =121; 9, N =485; 10, N =228; 13, N =50) during feeding. A series of nutritional trials such as forage feeding, concentrate rations or amino acid and mineral supplementation was imposed on 651 of the steers fed at the University of Missouri. Consequently, there were a total of 102 contemporary groups based upon nutritional trial, farm, year and season of origin represented in these data.
3. Hereford: 870 animals were individually fed a concentrate ration at Olsen Ranches, Inc in Harrisburg, Nebraska using a GrowSafe system. Olsen Ranches is the primary test herd for the American Hereford Association's National Reference Sire Program. Seedstock producers from around the U.S. nominate Hereford bulls for inclusion in the program. Phenotype and DNA samples were collected on 840 steers and 30 heifers born in Spring of 2009 (N =194), 2010 (N =293) and 2011 (N =383) and sired by 40 bulls with half-sib groups ranging in size from 1 to 104 and averaging 21.2 animals. Animals were fed for 70 (N =630), 72 (N =209) or 140 (N =31) days and had 8 (N =456), 9 (N =383) or 16 (N =31) weights recorded while on feed. A total of 10 contemporary groups based upon farm of origin, sex, feeding duration and slaughter date were represented in these data.
4. Simmental × Angus: 1,445 Simmental-sired steers originating from 6 ranches were individually fed concentrate rations at the University of Illinois at Urbana-Champaign using a GrowSafe system in 2005 (N =231), 2006 (N =320), 2007 (N =322), 2008 (N =347) and 2009 (N =225). The steers were produced from 122 registered Simmental bulls with half-sib groups ranging in size from 1 to 113 progeny and averaging 11.7. Steers were fed either 122 (N =225), 134 (N =50), 140–149 (N =270), 160 – 169 (N =274), 170 – 179 (N =417), 183 (N =57) or 195 days (N =152) and live weights were taken on two adjacent days at the beginning and at the end of each feeding period. A series of nutritional trials was imposed on these steers resulting in a total of 202 contemporary groups based upon nutritional trial, ranch and year of origin as well as slaughter group.

The Cycle VII animals were genotyped with the BovineSNP50 assay and data for 48,729 SNPs were analysed for this platform [37]. Animals from the Angus and Hereford populations were genotyped with both the BovineHD and BovineSNP50 assays with missing values imputed to the union of the marker sets using Beagle 4.0 with default parameters [83]. The 1,445 Simmental × Angus animals were genotyped with the BovineSNP50 assay, however, BovineHD data for 467 registered Simmental bulls were also available and were used for genotype imputation.

For the Angus, Hereford and Simmental × Angus datasets: Animals were removed from the dataset if their genotype call rates were less than 0.90, if their autosomal heterozygosity exceeded 45% or if they were predicted to be Klinefelter (XXY) individuals (N =7 Angus, N =2 Hereford). For animals genotyped with the 50 K assay (1,093 Angus, 361 Hereford, 1,445 Simmental × Angus), sex was assigned as male if non-pseudo-autosomal X (paX) locus heterozygosity was <0.03, otherwise the animals were assigned as female. For animals genotyped with the 770 K assay (510 Angus, 491 Hereford), sex was assigned as male if non-paX locus heterozygosity was <0.03 and Y locus call rate was ≥0.5, female if non-paX locus heterozygosity was ≥0.03 and Y locus call rate was <0.5, and Klinefelter if non-paX locus heterozygosity was ≥0.03 and Y locus call rate was ≥0.5. Hereford females (n =23) had BTA X heterozygosities (0.18) that were approximately one-half of their autosomal heterozygosities (0.32). The threshold of 0.03 was used to account for genotyping error rate. Similarly, BTA Y call rates were generally negligible in females and very high in males and the BTA Y threshold of 0.5 successfully identified the presence or absence of a Y chromosome. SNPs were removed from the dataset if they had a call rate <0.85, minor allele frequency <0.001 or Hardy-Weinberg Equilibrium p <3 × 10^−9^. Non-paX and BTA Y SNPs with heterozygosities >0.03 in males and mitochondrial SNPs with heterozygosities >0.03 were also removed leaving 747 473, 684 458 and 690 184 loci for analysis in the Angus, Hereford and Simmental × Angus populations, respectively.

For Angus and Hereford animals with weekly or biweekly body weight measurements and Simmental × Angus animals with two start and ending weights, ADG and MBW were estimated over the test period by linear regression. The total feed intake of each animal over the test period was averaged and adjusted for moisture content to produce the average daily DMI [7]. Residual feed intake was analysed by including partial linear regressions on ADG and MWT in the model used to analyse DMI.

### Statistical analysis

The BayesB method was used to simultaneously analyse whole genome SNPs, using GENSEL software [84]. For each of the 4 populations (Cycle VII, Angus, Hereford and Simmental × Angus), each trait (ADG, DMI, MBW and RFI) was separately analysed, with SNP allele substitution effects fitted as random effects. Systematic environmental effects fitted as fixed effects included cohort groups based on birth herd, year and season of birth and sex, resulting in 15, 102, 10 and 202 contemporary group levels in the Cycle VII, Angus, Hereford and Simmental × Angus populations, respectively. For the Cycle VII animals, linear covariates for breed composition and expected heterosis based upon the breed composition of each animal’s parents were also included in the model. The parameter π, which is the proportion of SNPs assumed to have no effect on the trait was set at 0.99 for the Cycle VII animals (genotyped with 50 K SNPs) and at 0.9995 for the other 3 populations (genotyped or imputed to 800 K SNPs) which corresponded to fitting about 400 markers in each MCMC iteration. Allowing only markers with strong associations to traits to be fitted, motivated the choice of π. MCMC methods with 41,040 iterations were used to generate posterior mean estimates of marker effects and variance components after discarding the first 1,000 samples for burn-in.

Due to linkage disequilibrium, the effect of a QTL may be spread over a number of neighboring SNPs. Therefore, the genome was divided into non-overlapping 1-Mb windows based on the UMD3.1 reference assembly base pair locations of markers and the percentage of genetic variance explained by each window was calculated for each trait. The null hypothesis distribution of the percentage of genetic variance explained by each 1-Mb window was generated for each trait by applying the same model (BayesB with the same parameters) on permuted data in the Angus population (data sets in which the genotypes of individuals are randomly assigned to trait values, which maintain the distributional properties of the trait values and the genotypes under the null hypothesis of no true QTL effects). As for the analysis of the unpermuted data, the genome was divided into the same non-overlapping 1-Mb windows and the percentage of genetic variance explained by each window was calculated. The JMP software [85] was used to fit the model and generate the distributions for the percentage of genetic variance explained by the 1-Mb windows. Next, the estimated parameters from the best-fit models (Table 6) were used to calculate the P-values for each window in the analysis of the unpermuted data. The estimated parameters for RFI were also used for ADG, as the estimated parameters for ADG were dissimilar to those for the other traits (Table 6) and produced spurious significance results. The Bonferroni correction was employed to adjust P-values for multiple test comparisons using the p.adjust package in R [86]. Windows with a Bonferroni-corrected P-value <0.05 were identified as significant QTL. The sfdp algorithm from Graphviz software was used to draw the QTL network [87].

**Table 6:** **Summary statistics and estimated parameters for the best-fit model (Johnson Su distribution) for the distribution of the percentage of additive genetic variance explained by 1-Mb windows generated from the permutated data in the Angus population** ^**1**^

Parameter	ADG	DMI	MBW	RFI
N	2,684	2,684	2,684	2,684
Mean	0.039	0.042	0.043	0.041
Standard deviation	0.017	0.048	0.054	0.045
−2Log(Likelihood)	−14,665.593	−12,985.369	−12,578.502	−13,090.396
Shape γ	−0.514	−1.019	−0.996	−1.001
Shape δ	1.772	0.924	0.908	0.976
Location θ	0.031	0.018	0.017	0.018
Scale σ	0.023	0.009	0.010	0.010

^1^ADG: average daily gain, DMI: dry matter intake, MBW: mid-test metabolic body weight, and RFI: residual feed intake.

Within each of the significant windows, the SNP with the highest sPPI (percentage of the chains in which the specific SNP is included in the model with non-zero effect) was chosen as the most strongly associated SNP within the 1-Mb QTL window and was denoted the ‘lead-SNP’. Posterior mean residual and additive genetic variances and posterior mean of marker-based heritability were reported for each trait in each population.

### Availability of data

The data sets supporting the results of this article are available for non-commercial purposes from JFT following the execution of a materials transfer agreement.

## Abbreviations

ADG: Average daily gain
BTA: *Bos taurus* chromosome
DMI: Dry matter intake
GWAS: Genome-wide association study
Mb: Million base pairs
MBW: Metabolic mid-test body weight
MCMC: Markov-chain Monte Carlo
paX: Pseudo-autosomal X chromosome
sPPI: Posterior probability of model inclusion for a given SNP
QTL: Quantitative trait loci
RFI: Residual feed intake
SNP: Single nucleotide polymorphism
